# Prominin-1 (CD133) Defines Both Stem and Non-Stem Cell Populations in CNS Development and Gliomas

**DOI:** 10.1371/journal.pone.0106694

**Published:** 2014-09-03

**Authors:** Karl Holmberg Olausson, Cecile L. Maire, Sam Haidar, Jason Ling, Emily Learner, Monica Nistér, Keith L. Ligon

**Affiliations:** 1 Center for Molecular Oncologic Pathology, Department of Medical Oncology, Dana-Farber Cancer Institute, Boston, Massachusetts, United States of America; 2 Department of Oncology Pathology, Karolinska Institutet, Stockholm, Sweden; 3 Department of Pathology, Boston Children's Hospital, Boston, Massachusetts, United States of America; 4 Department of Pathology, Brigham and Women's Hospital, Boston, Massachusetts, United States of America; 5 Department of Pathology, Harvard Medical School, Boston, Massachusetts, United States of America; Columbia University, United States of America

## Abstract

Prominin-1 (CD133) is a commonly used cancer stem cell marker in central nervous system (CNS) tumors including glioblastoma (GBM). Expression of Prom1 in cancer is thought to parallel expression and function in normal stem cells. Using RNA in situ hybridization and antibody tools capable of detecting multiple isoforms of Prom1, we find evidence for two distinct *Prom1* cell populations in mouse brain. *Prom1* RNA is first expressed in stem/progenitor cells of the ventricular zone in embryonic brain. Conversely, in adult mouse brain *Prom1* RNA is low in SVZ/SGZ stem cell zones but high in a rare but widely distributed cell population (*Prom1^hi^*). Lineage marker analysis reveals *Prom1^hi^* cells are Olig2+Sox2+ glia but *Olig1/2* knockout mice lacking oligodendroglia retain *Prom1^hi^* cells. Bromodeoxyuridine labeling identifies *Prom1^hi^* as slow-dividing distributed progenitors distinct from NG2+Olig2+ oligodendrocyte progenitors. In adult human brain, PROM1 cells are rarely positive for OLIG2, but express astroglial markers GFAP and SOX2. Variability of PROM1 expression levels in human GBM and patient-derived xenografts (PDX) – from no expression to strong, uniform expression – highlights that PROM1 may not always be associated with or restricted to cancer stem cells. TCGA and PDX data show that high expression of *PROM1* correlates with poor overall survival. Within proneural subclass tumors, high *PROM1* expression correlates inversely with *IDH1* (R132H) mutation. These findings support PROM1 as a tumor cell-intrinsic marker related to GBM survival, independent of its stem cell properties, and highlight potentially divergent roles for this protein in normal mouse and human glia.

## Introduction

Prominin-1 (Prom1, PROM1, CD133) is a pentaspan transmembrane glycoprotein originally identified in immature hematopoietic cells [1], [2] and now widely regarded as a marker of normal and cancerous stem cells particularly in the central nervous system (CNS) [3]–[7]. In the normal CNS, studies have primarily focused on characterization of Prom1 in stem cell compartments, but its expression in other cell types and their lineage is not well understood. Prominin-1 expression has been reported in oligodendroglia, ependymal cells, and in the human fetal spinal cord [8]-[10]. PROM1 cells isolated from the human fetal ventricular zone have the ability to generate neurospheres, which retain self-renewal and multi-lineage differentiation capacity [9].

In the adult brain, the distribution and characteristics of Prominin-1 cells are less well studied. Prom1 expression has been reported in ependymal cells and murine hippocampus [10], [11]. In transgenic Prom1-lacZ mice, Prom1/lacZ was co-expressed with Gfap in cells of the subventricular zone (SVZ) having properties of multi-potent self-renewing neural stem cells. However, Prom1/lacZ+Gfap- cells single-sorted from this region were not able to form secondary neurospheres or to differentiate into all neural lineages. LacZ expression was also noted in cells with non-stem cell phenotypes widely throughout the adult mouse brain in regions but whether the endogenous gene is expressed in a similar pattern was not fully established [5], [12].

PROM1 is believed to identify tumor-initiating cancer stem cells in a wide range of cancer types including leukemia [4], breast [3] and glioblastoma (GBM), the most common malignant brain tumor [13]. The cancer stem cell hypothesis suggests that only a minor subpopulation of the tumor cells maintain tumor growth and have the indefinite capacity to self-renew. Based on flow cytometry analysis, PROM1 cells in GBM have been described as tumor initiating cells able to propagate tumor growth in xenograft models and confer radioresistance [7], [14], [15]. However, GBM PROM1 negative cells can also contribute to tumor propagation [16], [17]. This raises the possibility that PROM1 may not be as closely linked with “stemness” or tumor initiating phenotype in normal cells or cancer cells as previously proposed [18].

Recent studies of Prominin-1 expression have used alternatives to flow cytometry, which allow more direct in situ visualization of Prom1 expression. Such studies increasingly describe differences in expression of the multiple complex Prom1 isoforms in mouse and human. These have also increasingly highlighted its non-stem cell functions in the hematopoietic, retinal and prostate systems [19]–[21]. In addition, expression of Prominin-1 has been reported as regulated by hypoxia, supporting the possibility that Prominin-1 may be a dynamic marker not necessarily associated with cell lineage or stemness phenotypes [22]. Considering these findings we sought to further explore the normal and cancer expression pattern and lineage associations of Prominin-1 in human and mouse brain.

## Materials and Methods

### Animals

Animal husbandry was approved and performed according to Dana-Farber Cancer Institute's animal care and use policies. The Institutional Animal Care and Use Committees of Dana-Farber Cancer Institute specifically approved protocol 09-016 that covered all animal procedures.

### Lineage analysis

100 *Prom1^hi^* cells were counted for each animal and four animals were used for each marker analysis. Pictures were taken on coronal sections of the forebrain from the lateral ventricle to the hippocampus, with SVZ and corpus callosum included. Results are presented as the mean calculated between different animals.

### BrdU labeling

5-bromodeoxyuridine (BrdU, Sigma) was administered as a single dose of 50 mg/kg 2 h before euthanasia for short term labeling or was given once daily for 7 and 14 days for long term labeling. Two animals were used per time point and about 100 cells expressing BrdU were counted for each area per animal. Pictures were taken on coronal sections of the forebrain from the lateral ventricle to the hippocampus, with SVZ and corpus callosum included.

### Stab wound lesion

Stab wound lesions were performed at the following coordinates (1.5 mm, 3 mm and 2.5 mm). This was repeated 3 times creating 4 stab lesions moving 1.5 mm posterior between each lesion. *Prom1^hi^* and Olig2 expressing cells were quantified on 10 pictures (40x). The contra lateral side was used as control. Results are presented as the mean calculated between different animals; two animals were used for each time point.

### Irradiation

Balb/c mice were given a dose of 3 Gy and sacrificed at 24 h and 5 d. The number of *Prom1^hi^* cells was determined on coronal sections of control and irradiated brains from the lateral ventricle to the hippocampus. Results are presented as the mean calculated between different animals and two animals were used for each time point.

### Quantitative real time PCR

Specific brain regions were dissected under a dissecting microscope from 300 um thick fresh vibratome sections, and each region studied consisted of pooled tissue from 3 mice. *Prom1* (Mm 00477115_ml) was analyzed alongside *Olig2* (Mm 01210556_ml) with *beta-actin* (00607939_sl) as control. This experiment was done twice.

### MACS and FACS

Dissected brain regions were dissociated using Neural Tissue Dissociation Kit from Miltenyi Biotech. Cell suspensions were processed using MACS anti-Prominin-1 magnetic micro-beads (Miltenyi) or Percoll gradient to remove the myelin debris. For FACS sorting, cell suspensions were incubated with anti-Prominin-1 PE conjugated antibody 1∶20 (Miltenyi, AC133). Samples were analyzed using BD FACS Aria II.

### GBM patient derived cell lines (PDCL)

Patient samples used for this research were collected under two waived consent protocols, Dana Farber Harvard Cancer Center protocol #10-043 and Partner's Human Research Center protocol #2002 P000995, as well as an informed consent protocol under Dana Farber Harvard Cancer Center protocol #10-417. All protocols mentioned have been approved by Dana Farber Harvard Cancer Center and Partner's Human Research Center institutional review boards. Patient derived cell lines were established from GBM resections, newly diagnosed (n = 17) and recurrent samples (n = 5). Tissue was disaggregated using the gentleMACS Dissociator and enzymatically digested with the Neural Tissue Dissociation Kit with Papain (Miltenyi). Cells were plated at a density of 500,000 to 1,000,000 cells in Corning Ultra-Low attachment cell culture flasks 75 cm^2^. Cells were cultured in neural stem cell media as neurospheres in Stemcell Technologies NeuroCult NS-A Proliferation Kit (Human) supplemented with 2 ug/ml Heparin, 20 ng/ml human EGF, and 10 ng/ml human bFGF. Cells were fed weekly by adding fresh media with growth factors.

### GBM patient derived xenografts (PDX)

Cells were dissociated mechanically and 100,000 cells in 1 ul were injected intra cranially in IcrTac:ICR-Prkdc^SCID^ mice (2 mm; 0 mm; 2 mm).

### Tissue Fixation

Mice were perfused with 4% paraformaldehyde (PFA) in PBS (pH 7.0). Brains were post fixed overnight in 4% PFA, cryoprotected using 0.5 M sucrose/PBS and embedded in OCT compound (Tissue-Tek). Frozen sections were sectioned at 12 um (CM1850, Leica) and harvested on superfrost plus glass slides (Fisher).

### RNA In Situ Hybridization (RISH)

Frozen sections were heat treated, 30 min 90°C in 10 mM Sodium Citrate buffer pH 6.0 in a steamer, for antigen retrieval. Sections were pre-hybridized using hybridization buffer followed by overnight hybridization with digoxigenin (DIG)-UTP-labeled RNA-probe (1∶500 in hybridization buffer). The probe was synthesized using a DIG labeling kit (Roche). The plasmids mCD133-pCMV-SPORT6, Open Biosystems; #11776, BC028286 (mouse PROM1) linearized with Sma1 and hCD133-pOTB7, hORFeome; MHS1011-76019-4644690, BC012089 (human PROM1) linearized with BamH1 were used as templates and transcribed using T7 RNA polymerase to create antisense RNA. Detection was done with sheep anti-DIG Fab fragments conjugated with alkaline phosphatase (Roche). BM Purple (Roche) was used to visualize bound probe by overnight incubation. For double labeling by RISH/IHC, sections were stained, as described below, after the BM purple development step of the RISH (not repeating antigen retrieval).

### Immunohistochemistry (IHC)

Frozen sections were subjected to antigen retrieval in 10 mM citrate buffer and blocked using peroxidase-blocking reagent (DAKO). Primary antibodies were incubated overnight at 4°C, secondary antibodies were incubated for 2 hours at room temperature and then revealed using horseradish peroxidase-DAB (EnVision Doublestain Kit, DAKO; K1395). The following antibodies were used: mouse monoclonal anti-human Prom1/CD133 1∶100 (Miltenyi; W6B3C1) expected to recognize the human CD133/1 epitope (same as AC133), rat monoclonal anti-mouse Prom1/CD133 1∶100 (eBioscience; 13A4), rabbit polyclonal anti-mouse/human Olig2 1∶10000 (Chemicon; AB9610), rabbit polyclonal anti-mouse/human Sox2 1∶100 (Chemicon; AB5603), mouse monoclonal anti-mouse/human NeuN 1∶100 (Chemicon; MAB377), rabbit polyclonal anti-mouse/human GFAP 1∶2000 (DAKO, Z0334), mouse monoclonal anti-BrdU 1∶100 (BD; 347580), rabbit polyclonal anti-mouse NG2 1∶100 (Chemicon, AB5320).

### Immunofluorescence (IF)

Frozen sections were subjected to antigen retrieval in 10 mM citrate buffer then blocked in 10% goat serum, and incubated with primary antibody over night at 4°C, washed in TBST, incubated with secondary antibody and DAPI for 2 h at room temperature. The following primary antibodies were used: mouse monoclonal anti-human PROM1/CD133 1∶100 (Miltenyi; W6B3C1), rabbit monoclonal anti-human SOX2 1∶1000 (Cell Signaling technologies; 5024), rabbit polyclonal anti-mouse/human Olig2 1∶1000 (Chemicon: AB9610), rabbit polyclonal anti-mouse/human GFAP 1∶200 (DAKO; Z0334), rabbit polyclonal anti-human GFAP-Delta 1∶500 (Abcam; ab28926), rabbit polyclonal anti-human Ki67 1∶1000 (Novocastra; NCL-Ki67p). Secondary antibodies were anti-mouse Alexa 568 1∶1000 (Invitrogen), anti-rabbit FITC 1∶100 (Zymed).

### Western blot

Tissues were dissected under a dissecting scope from 300 um thick vibratome sections. Cell lines were pelleted and frozen at −80°C. Proteins from tissue and cells were extracted in RIPA buffer (Boston BioProducts). Equal amounts of protein (20 ug) were loaded onto the gel (Invitrogen 4–12% Tris-glycine gel). Samples were transferred, using BioRad semi dry transferring device at 110 V for 1 hour, to Invitrogen Nitrocellulose membrane and blocked over night at 4°C in 5% milk/PBS. The membrane was washed in PBST and incubated with primary antibody/PBST for 1 hour. After washing in PBST, the membrane was incubated with secondary HRP coupled antibody for 1 h at room temperature and revealed using HyGlo solution (Denville scientific). Antibodies used were rat monoclonal anti-mouse Prom1/CD133 1∶100 (eBioscience; 13A4), for mouse proteins and mouse monoclonal anti-human PROM1/CD133 1∶100 (Miltenyi; W6B3C1) for human proteins. GAPDH (1∶1000, Cell Signaling Technologies) was used as loading control. Secondary antibodies were goat anti rat-HRP 1∶2000 (Zymed) and rabbit anti Mouse-HRP 1∶2000 (Dako EnVision).

### Expression profile analysis

We obtained gene expression profile data from The Cancer Genome Atlas (TCGA) data [23], [24] and PROM1 probe value on our cohort of cell cultures established from GBM samples was obtained using HG U133 Plus 2.0 array (Affymetrix) and presented in table S1. The PROM1 probe is identical in both array platforms (204304_s_at). For analyses performed we binned all samples into high (mean value >1000) and low (mean value <300) PROM1 expression groups. Fold change of the mean expression probe value and a p-value was calculated using two-sided Student's t-test. The cBioPortal [25], [26] was used to analyze TCGA GBM data regarding PROM1 expression, PDGFRA aberrations, IDH1 mutation [27] and patient survival [23].

## Results

### Prom1 RNA is highly expressed in neural stem and progenitor cell zones in prenatal development

Previous studies of Prom1 expression have been challenging to interpret due to the fact that many splice variants and modified forms of Prom1 protein exist, and the exact forms recognized by different antibodies have not been clearly defined. We therefore performed an RNA in situ hybridization (RISH) study of mouse development using a mouse *Prom1* probe derived from the open reading frame (ORF) of the Prom1 gene [28] to detect as many splice variants as possible.

**Figure 1:**
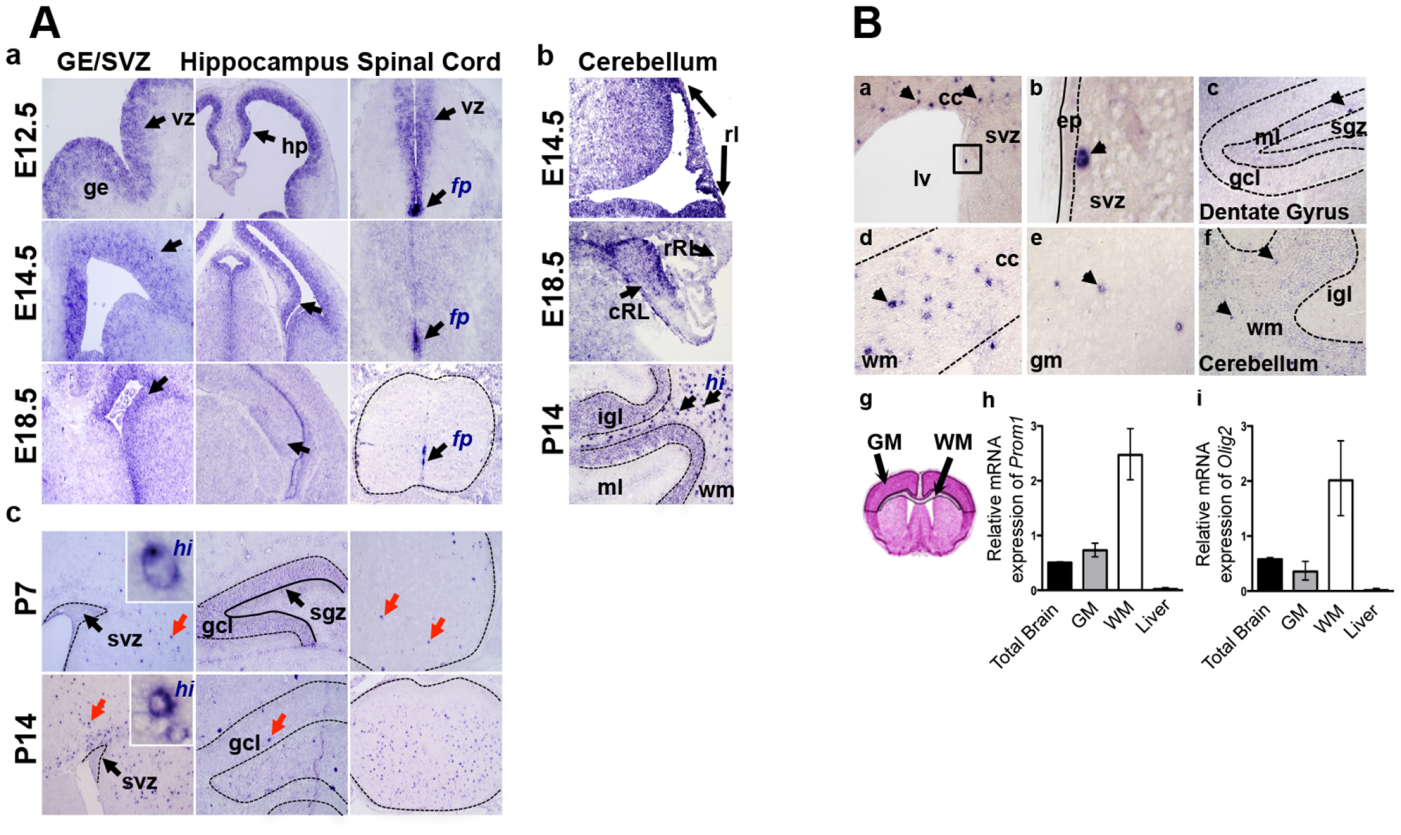
*Prom1* expression in developing and adult mouse CNS. A. RNA in situ hybridization showing high *Prom1* expression in the ventricular zone (vz, arrows) of the prenatal brain and spinal cord (a). Levels decrease over time except in the floor plate (fp). In the cerebellum (b) *Prom1* is highly expressed in the caudal and rostral rhombic lips (rl, cRL, rRL) while postnatal expression is most intense in rare cells of the white matter (hi, arrows) and the internal granule layer (igl). In postnatal brain (c) expression is conserved at low levels in the stem cell niches (svz and sgz) but is highest in single cells within white matter (wm, red arrows). B. In adult brain *Prom1* RNA is highest in single, widely distributed cells (arrowheads) in the corpus callosum (a, d), cerebellum (f), gray matter (e) and only rarely in SVZ (b) and the SGZ (c) stem cell niches. Semi-quantitative RT-PCR of microdissected white and grey matter regions from 3 different mice (g) validate high *Prom1* levels in white matter relative to gray matter and whole brain (p = 0.05) (h). Mouse liver acts as a low level reference tissue. The differential level of *Prom1* is similar to the oligodendroglial marker *Olig2* (i). ge: ganglionic eminence; ml: molecular layer; gm: grey matter; hp: hippocampus; gcl: granule cell layer; sgz: subgranular zone; cc: corpus callosum; lv: lateral ventricle; ep: ependymal layer.

*Prom1* was expressed in all ventricular zone regions of the central nervous system from E12.5–18.5 (Fig. 1A–a,b). *Prom1* was noted in the ganglionic eminence, hippocampal primordium, spinal cord and cerebellar ventricular zone and rhombic lip regions throughout this period. There was little evidence of significant anatomic or subdomain restriction, although the floor plate in the spinal cord appeared to maintain high levels of *Prom1* up until birth as previously reported [9]. Expression was low to absent in more differentiated cells adjacent to, or outside the ventricular zone regions and progressively decreased with time in all regions. The rapid downregulation of *Prom1* in all regions and cell types suggested that *Prom1* expression is highly restricted to undifferentiated radial glial progenitors and their immediate progeny during prenatal development.

### Dynamic changes in Prom1 expression occur in early postnatal development

Examination of early postnatal developmental stages showed a dynamic switch in the *Prom1* pattern compared to the prenatal period. Beginning at P0–P7 high levels of *Prom1* were detected within rare single cells distributed throughout the developing white matter and cortex (*Prom1^hi^*) (Fig. 1A,c). These cells appeared distinct from the prenatal *Prom1* cells in that expression levels were higher and the appearance of such cells in each anatomic region was generally not associated with the maturing stem/progenitor cell zones. The *Prom1^hi^* cells noted throughout all the regions of the CNS had the same appearance as those reported previously in the cerebellar white matter [8]. The *Prom1^hi^* cells did not appear to represent maintenance of expression within cells migrating out of the stem cell zones due to much lower expression in regions immediately adjacent to these zones and their widespread distribution. *Prom1* expression in maturing ventricular zone regions during P0–P14 progressively declined and was in fact was extremely low within the specific stem cell niches of the SVZ and SGZ, where it was expected to be highest based on previous reports (Fig. 1A c). During this same period several differentiated cell types appeared by morphology to express low levels of *Prom1* including the dentate gyrus granule cell neurons of the hippocampus, the internal granule cell layer neurons of the cerebellum, and the ependymal lining of the ventricles (Fig 1A c).

### Prom1 is expressed in a rare but widely distributed cell population in adult mouse brain

The overall expression pattern in adult mouse brain was similar to that seen in the early postnatal period. The *Prom1^hi^* cells persisted as a rare widely distributed population throughout the entire brain and was considered the predominant cell population expressing high levels of the gene (Fig. 1B). *Prom1^hi^* cells were more frequent in white matter tracts than in grey matter regions (Fig. 1B a,d,e). Low level diffuse *Prom1* expression was noted in the hippocampal marginal layer and cerebellar white matter but was not apparently associated with Bergmann glia (Fig. 1B c,f). Occasional *Prom1^hi^* cells were located within or near the SVZ and SGZ (Fig. 1B a,b,c, arrows) possibly representing overlap with previously described activated neural stem cells [29] but did not appear to occur at same frequency expected based on recent reports [29], [30]. Very low diffuse levels of *Prom1* were detected in adult ependymal/subependymal cells (Fig. 1B b), although such results were variable presumably due to being at the limit of detection.

To validate the RISH data, we performed qRT-PCR on micro-dissected regions from vibratome sections of adult mouse brain. We found that white matter contained more than 3 times higher levels of *Prom1* than grey matter, similar to expression of the oligodendroglial lineage gene, *Olig2* (Fig. 1B g,h,i). Total brain and dissected sub-regions contained 0.5–2.5 fold more RNA overall than mouse liver, an organ previously noted to contain low levels of *Prom1* [6].

Given that most prior studies of Prom1 protein did not observe high levels in white matter regions, we independently evaluated the presence of Prom1 protein by IHC and Western blot (Fig. S1). Many Prom1 antibodies are reported to recognize specific glycosylated antigens (e.g. AC133, CD133) of the Prom1 protein and we chose to use an antibody that recognizes both glycosylated and unglycosylated forms of Prom1 (eBioscience; clone 13A4) [31]. This antibody is expected to recognize all splice forms except s4 and s5 [6] but would appear to be more restricted compared to our RNA probes. We noted that Prom1 protein in adult brain was generally low, but most readily detected in cilia of ependymal cells lining the lateral ventricle (Fig. S1A). Protein staining in SVZ and SGZ was generally low relative to ependyma. Interestingly, no positive cells were detected by IHC in the parenchymal white matter or grey matter regions that could be regarded analogous to the *Prom1^hi^* cells detected by RISH. Diffuse weak signal was sometimes seen in white matter and neuropil regions consistent with either weak staining or background but was also not consistently detected (see discussion).

Given the differences in the RISH and IHC staining patterns, we hypothesized that the protein and its glycosylated or other forms may not be fully recognized in the IHC analysis. We therefore performed FACS analysis to determine native Prom1 protein levels. Dissected adult brain regions containing grey matter (neocortex) or white matter (corpus callosum), were incubated with Prom1 antibody. Positive and negative fractions were obtained by MACS and subsequently quantified by FACS. For the white matter tissue isolation we first used a Percoll gradient to clean up the myelin debris from white matter cells and then performed FACS directly on the total cell population (MACS was not possible as an initial enrichment step due to the fibrous and sticky nature of myelin). Prom1 positive cells comprised 83% of the white matter cells versus only 22% of the grey matter cells suggesting 4 fold higher numbers of Prom1 positive cells in white matter, similar to results for white and grey matter ratios seen with RISH and qRT-PCR analysis (Fig. S1D, E). To further delineate the pattern of Prom1 protein, we performed western blot of micro-dissected regions including grey matter, white matter, ependyma, and SVZ. Prom1 was detected as a 100 kDa band in every region, with the highest signal in white matter and low levels in the SVZ/ependymal layer (Fig. S1B, C). In conclusion, we find that both Prominin-1 RNA and protein appear to be highest in a widely distributed cell population in grey and white matter of the adult mouse brain, with much lower levels in the SVZ/ependymal regions.

### Prom1^hi^ cells are glial cells with unique properties

**Figure 2:**
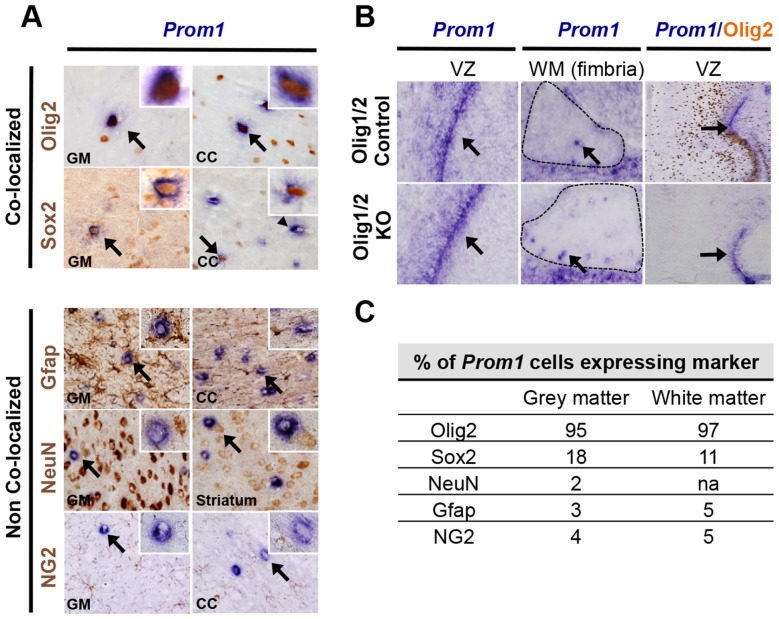
*Prom1^hi^* cells are glial cells with novel characteristics. A. Combined RISH:IHC shows single cells with high *Prom1* RNA (blue, ISH) co-localize with oligodendroglial marker Olig2 and astroglial marker Sox2 (brown, IHC) in both white and grey matter. No co-localization was detected with Gfap, NG2 or NeuN. B. Olig1/2^−/−^ knock-out mice (Olig1/2 KO) show *Prom1* staining in ventricular zones (arrows) and fimbria white matter where *Prom1^hi^* cells first emerge (arrows) in E18.5 mouse brain. Olig2+ cells retain protein in Olig1/2 controls (brown, IHC). C. Quantification of the *Prom1* RISH cells co-localizing with lineage markers. cc: corpus callosum; gm: grey matter, wm: white matter; vz: ventricular zone.

To determine the cell lineage of the distributed *Prom1^hi^* cells in the adult mouse brain, we performed double labeling with *Prom1* RISH and IHC for developmental lineage markers (Fig. 2). Nearly all *Prom1^hi^* cells co-stained with the progenitor cell and oligodendroglial lineage marker Olig2 in the grey and white matter (Fig. 2A, C 95–97%). In addition, a subpopulation of *Prom1^hi^* cells stained positive for the stem cell/astrocyte transcription factor Sox2 (Fig. 2A, C 11–18%). Only rare weakly *Prom1* positive cells showed possible co-localization with Gfap (astrocytes, 3–5%), NeuN (neurons, 2%) or NG2 (oligodendroglial progenitors, 4–5%) (Fig. 2A, C).

Prior studies have suggested that Prom1 is expressed in mature myelinating oligodendrocytes [32], however the rarity of *Prom1^hi^* cells and heterogeneity with respect to Sox2 expression observed here are not typical for myelinating oligodendrocytes or other known subclasses of oligodendroglia. To address whether *Prom1^h^* ^i^ cells are myelinating oligodendroglia we examined *Prom1* expression in Olig1/2 deficient mice in which the entire oligodendroglial lineage including immature and mature myelinating oligodendroglial cells, fail to develop (Fig. 2B) [33]–[35]. Surprisingly, at E18.5, the latest stage possible to examine in this genetic background due to embryonic lethality associated with the lack of oligodendrocytes, *Prom1* expression in the ventricular zone of knockout mice was retained and appeared identical to controls. In addition, rare *Prom1^hi^* cells in the fimbria, one of the earliest white matter tracts to undergo myelination in normal mouse development, were also unaffected compared to controls (Fig. 2B). This suggested that *Prom1^hi^* cells are not restricted to mature myelinating oligodendrocytes but also represent a unique glial cell type not dependent on Olig gene function.

### Prom1^hi^ cells are slowly-dividing

**Figure 3:**
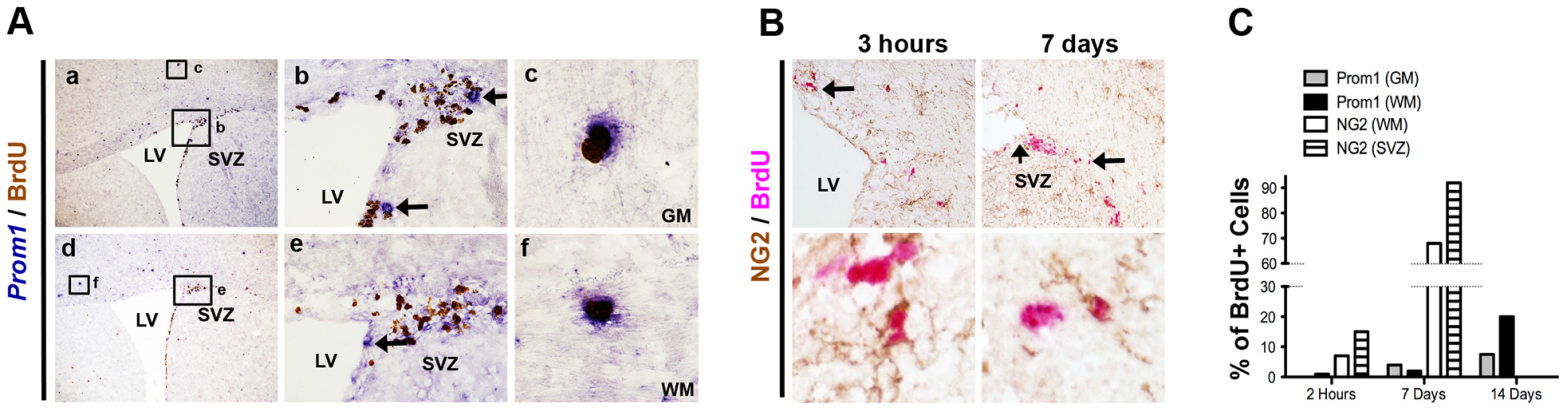
*Prom1^hi^* cells are slow dividing distributed progenitor cells distinct from adult NG2 progenitors. A. Two examples of long term BrdU labeling (7 days, daily dosing) identify rare Prom1+ (RISH; blue) BrdU+ (IHC; brown) cells in white and grey matter (c and f are magnified insets of the small marked area in a and d). High magnification of the SVZ (b, e) shows numerous BrdU+ stem/progenitor cells that do not express high *Prom1*. Arrows indicate rare Prom1^hi^+ cells that do not express BrdU (b, e). B. In contrast to Prom1, double IHC for NG2 (brown) and BrdU (red) readily identifies numerous co-localized cells in SVZ and brain parenchyma. C. Manual quantification of the percentage of BrdU positive cells co-labeled with *Prom1* or NG2 in the white matter, grey matter and SVZ. svz: subventricular zone; lv: lateral ventricle; gm: grey matter; wm: white matter.

To evaluate whether *Prom1^hi^* cells might represent a cycling progenitor cell population and to determine the kinetics of their turnover, we administrated BrdU to normal adult mice (Fig. 3). Incorporation of BrdU 2 hours after a single dose, or 7 and 14 days of daily doses was robust within the SVZ region but the rare *Prom1^hi^* cells in this region were not co-localized with BrdU (Fig. 3A b, e). In contrast, rare BrdU positive cells co-expressed *Prom1* in grey and white matter regions (<2% of total BrdU cells) 2 hours after a single BrdU administration. Co-labeled cell number increased following 7 and 14 days of daily BrdU administration with the percentage of BrdU positive cells expressing *Prom1* being 4 and 20% respectively (Fig. 3A c,f, C). This slow rate of incorporation in *Prom1^h^* ^i^ contrasted with the finding that by 7 days 68% of BrdU positive cells co-expressed NG2, a marker of the most abundantly distributed progenitor cell population in the adult brain (Fig. 3B, C). Moreover, *Prom1^hi^* cells in grey and white matter (corpus callosum) appeared unaffected by irradiation suggesting that such cells are radioresistant relative to the rapidly cycling transit amplifying cells of the SVZ (Fig. S3). While not fully quantitative, overall these data qualitatively support that *Prom1^hi^* cells scattered in the white matter and grey matter are slowly-dividing glial progenitor cells within the adult mouse brain and appear to be distinct from the more rapidly dividing larger population of NG2 positive distributed progenitor cells.

### Human Prominin-1 expression and cell lineage association differ from mouse

**Figure 4:**
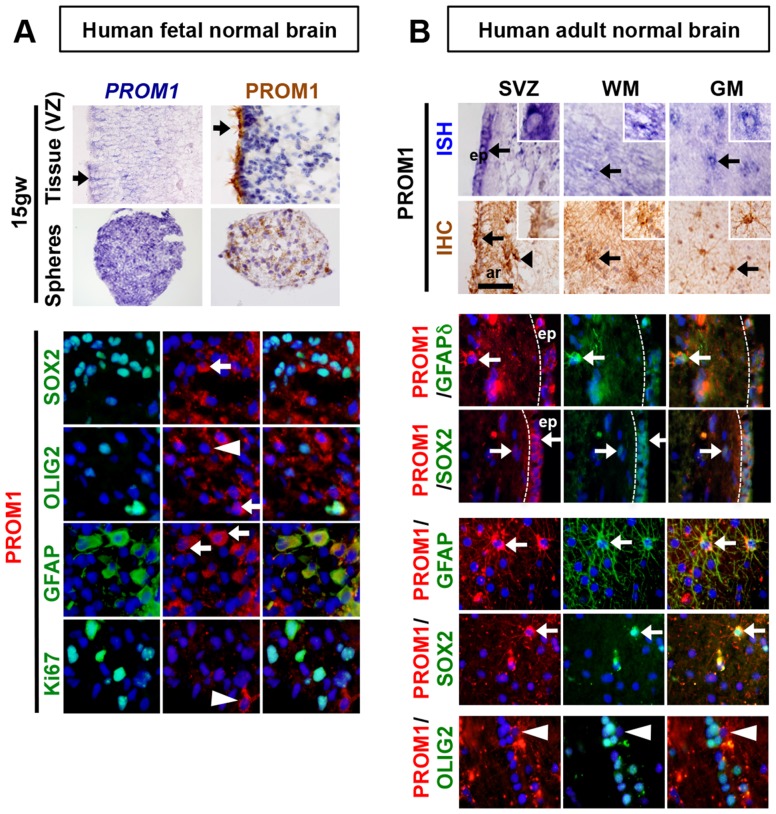
PROM1 is expressed in human fetal neural stem cells and in two distinct cell populations in adult human brain. A. PROM1 RNA and protein are highly expressed in ependymal/subependymal cells of the ventricular zone (arrow) particularly in ciliated cells. Images are from the anterior-lateral germinal matrix/SVZ in the developing cortex. A more heterogeneous expression of PROM1 is detected in neurospheres derived from human fetal brain (15 gw). Immunofluorescence lineage analysis shows co-localization of PROM1 with SOX2 and GFAP (arrows) but only rare co-localization with OLIG2 (arrowhead). Co-localization was not detected with proliferating Ki67 positive cells (arrowhead). B. RISH and IHC analysis, on human adult normal brain tissue shows high PROM1 expression in ependymal cells (arrows) and in cells with glial morphology with complex processes dispersed within the white and grey matter regions (arrows). PROM1 is present in ependymal cells (ep) and cells within the subependymal astrocyte ribbon (ar) also positive for GFAPδ and SOX2. White and grey matter regions exhibit PROM1 cells with astrocytic morphology that co-localize with markers SOX2 and GFAP (arrows). PROM1 and OLIG2 co-localization was not detected (arrowhead).

Given the differences between mouse and human Prominin-1 structure we sought to more carefully evaluate PROM1 expression and lineage associations in normal human brain, where its *in situ* expression is not well characterized. In human fetal brain (15 weeks estimated gestational age) there was weak *PROM1* RNA expression in the stem/progenitor containing ventricular zone (VZ) and ependymal regions, most prominently seen in what appeared to be an interdigitated subependymal cell population. By immunohistochemistry, PROM1 protein staining was highest in the microvilli and cilia of ependymal and/or subependymal cells. Cell bodies of ependymal/subependymal cells were not strongly stained (Fig. 4A).

To evaluate PROM1 expression in hNSCs distinct from the ependymal/subependymal cells, we isolated NSC containing neurospheres from 15 weeks gestational age human fetal brain ganglionic eminence, and stained the sectioned spheres under growth conditions for PROM1 RNA and protein. Broad distribution of the RNA and a more restricted distribution of protein were noted. Immunofluorescence co-staining of spheres showed PROM1 co-localized with the glial markers GFAP (95%), SOX2 (80%), and to a lesser degree OLIG2 (15%). However, the high percentage of GFAP+ cells in human fetal neural stem cell cultures may be affected by cell culture conditions given that endogenous GFAP expression appears somewhat later in development in the, 20 to 23 weeks gestation, human ventricular zone [36], [37]. No definitive PROM1+Ki67+ cells could be identified suggesting PROM1 cells were in a differentiated or a non-dividing/quiescent stem cell state (Fig. 4A).

In the adult human brain, PROM1 RNA and protein were high in the ependyma and cilia, similar to prenatal stages (Fig. 4B). PROM1 RNA and protein were also strongly expressed in cells with a glial morphology (complex processes by IHC) in the subependymal astrocyte ribbon and corpus callosum, possibly representing overlap with recently reported activated SVZ stem cell subpopulations [29], [38]. By immunofluorescence analysis we noted PROM1 cells in the subependymal astrocytic ribbon co-expressing the human neural stem cell marker GFAPδ [39], but no significant co-localization with either SOX2 or OLIG2 was detected in the sub-ventricular zone proper. Rare widely distributed PROM1 cells were detected in the adult white matter and subpial zones similar to *Prom1^hi^* cells in the mouse [32]. However, human *PROM1^hi^* cells stained positive for the mature astrocyte markers GFAP and SOX2 but not OLIG2 (Fig. 4B). Adult human PROM1 expression therefore differs from mouse and is predominantly associated with cells having an astrocytic phenotype.

### Visualization of PROM1 expression in purified GBM cells

Given our findings that PROM1 is expressed in stem as well as non-stem cells during normal development of mouse and human we sought to examine whether human GBM PROM1/CD133+ cells might have more similarities to the *PROM1^hi^* cells versus neural stem cells. To avoid analysis of PROM1 expressing normal cell types within primary tumors (e.g. vascular and inflammatory cells) we first examined PROM1 expression in “pure” GBM cell line models (PDCLs) and xenografts (PDXs).

**Figure 5:**
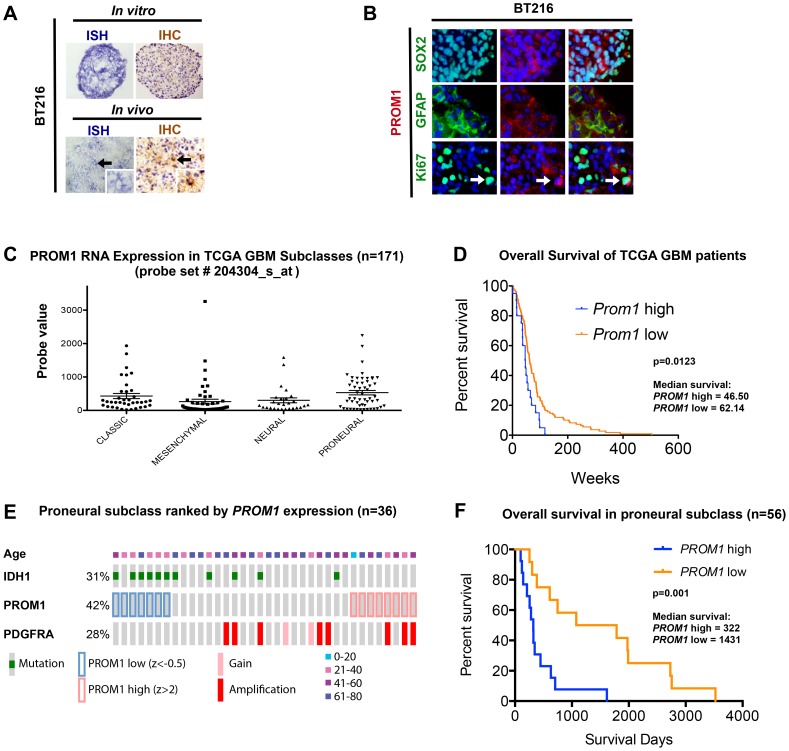
High PROM1 expression is associated with IDH1 wild-type, proneural GBM patients with poor survival. A. PROM1 RISH and IHC show similar patterns of expression *in vitro* (PDCL; BT216) and *in vivo* (PDX; BT216). B. Immunofluorescence on BT216 PDCL shows PROM1 co-localization with GFAP and SOX2 but rarely with proliferative marker Ki67 (arrow). C. *PROM1* RNA expression is highest in TCGA proneural subclass, not statistically significant. *PROM1* ^high^ cases are found in all 4 groups. D. *PROM1* high expression correlated with shorter overall survival in GBM patients from TCGA. P values using Mantle-Cox. E. cBioPortal analysis of the proneural TCGA subclass shows that *PROM1* ^low^ correlates with IDH mutation, while *PROM1* ^high^ is more associated with PDGFRA amplification. F. Kaplan-Meier analysis of *PROM1* ^high^ and *PROM1* ^low^ cases from the TCGA proneural subclass.

Immunohistochemical analysis of a panel of 22 patient-derived GBM cell lines grown *in vitro* under neural stem cell conditions (Fig. S4A) showed that 59% of lines (13/22) exhibited very few (<20% of cells) or no PROM1+ cells and intermediate staining (positivity in 20–50% of cells) in 18% of the lines (4/22). However 23% of PDCLs (5/22) had expression in nearly all tumor cells (>90% of tumor cells). These findings were confirmed in a subset of lines by flow cytometry (Fig. S4B). Similarly, PROM1 levels were preserved in PDX derived from these same lines (Fig. 5A). Immunofluorescence studies of PDCLs and PDXs (n = 5) revealed that PROM1 and the proliferation marker KI67 (non-G0 cells) generally showed only a rare degree of overlap (Fig. 5B and S5). Analysis of lineage markers showed a pattern similar to normal PROM1 glial cells with preferential, but not exclusive, expression of the astrocytic markers GFAP and SOX2. Very few PROM1 positive GBM cells co-expressed the oligodendroglial marker OLIG2 (Fig. S5).

### PROM1 expression is correlated with shorter survival in GBM

Prior work suggests that higher PROM1 expression in primary GBM samples may be associated with poor survival. We examined the distribution of *PROM1* RNA expression across all subclasses of TCGA samples [23] and our PDCL library, binning patients into high (>1000 units), medium (300–1000 units), and low (<300 units) groups based on absolute expression values (Fig. S6A, Table S1). Median patient survival was significantly lower in tumors with high *PROM1* expression compared to those with low expression in both the TCGA (p = 0.0123) and PDCL (p<0.0001) datasets (Fig. 5D and Fig. S6B).

Absolute *PROM1* RNA levels in 171 primary GBM patient samples from the TCGA showed that the proneural subgroup had the highest average expression, significantly higher compared to the mesenchymal subgroup (ANOVA p = 0.0239 followed by Tukey's multiple comparison test) (Fig. 5C). Moreover, patients with the highest expression of PROM1 (>1000 units) are predominantly found in the proneural subclass (12 out of 23).

More in depth analysis of the TCGA proneural subclass showed that *PROM1* ^low^ (Z score <−0.5) cases were enriched for IDH1 (R132H) mutation [27]. In contrast, *PROM1* ^high^ (Z score >2) cases showed no IDH1 mutations and were enriched for PDGFRA amplification, which has been associated with poor survival in the proneural subclass [40] (Fig. 5E and Fig. S6C). In the proneural group, *PROM1* ^high^ and *PROM1* ^low^ samples showed a dramatic split in the Kaplan-Meier curves where *PROM1* ^high^ correlates negatively with overall survival and disease-free survival (Fig. 5F) and showed no correlation with age (Fig. 5E). This establishes *PROM1* as a tumor cell intrinsic determinant associated with differential patient survival.

## Discussion

Examination of Prominin-1 RNA and protein *in vivo* across the entire CNS at embryonic through adult stages fully establishes Prom1 as a neural stem cell marker present throughout the ventricular zone of both the spinal cord and brain. Its expression pattern in these zones during prenatal development is diffuse and generally not restricted to specific progenitor subdomains known to exist in these regions [41], [42] with the exception of the floor plate of the neural tube [9]. The absence of staining in early, intermediate or migrating progenitors suggests that *Prom1* is rapidly down regulated and precisely tied to the differentiation state of stem cells. This pattern appears to be conserved in the early human VZ and stem cells cultured from this region, but the human PROM1 expression pattern was more difficult to comprehensively assess due to the weak expression and the limited samples available. Collectively however the pattern suggests Prominin-1 may have a shared stem cell function in human and mouse CNS at early embryonic developmental stages.

In the transition from embryonic to perinatal and adult stages of development we identified a clear and dynamic shift in Prom1 expression. Prior investigations at postnatal time points have largely focused on expression in stem cell niches or progenitor zones of the VZ, SVZ, SGZ, and ependyma but rarely describe Prom1 away from these zones [11], [12]. Our studies confirm expression in progenitor regions and *Prom1^h^* ^i^ cells as reported by others and possibly those cells identified recently as functional stem cells expressing GFAP and EGFR in a recent study of adult mouse SVZ [29], [30]. Such cells were also possibly identified in the human SVZ in our studies. However, using our methods, Prom1 is remarkably low relative to the levels of RNA detected in *Prom1^hi^* cells of the grey and white matter, unassociated with any stem cell niches. It is important to note however that discrepancies between the PROM1 mRNA and protein expression have been reported before in human colon tissue mostly due to difference in glycolyslation of CD133 [43]. The non-niche *Prom1^hi^* cells appear to be identical to cells previously identified as “lineage null” neural stem cells in the early postnatal cerebellar white matter [8] but highlights that they are in fact present throughout the entire CNS at all postnatal and adult stages.

Previous reports suggested that the *Prom1^hi^* cells isolated from the perinatal (P7) cerebellum could be cultured as multipotent neural stem cells [8]. However whether such cells might also exist in adult cerebellum or other regions of the brain has not been entirely clear. As part of our own investigations, we tested whether NSC cultures could be isolated from adult Prom1 positive cells by FACS sorting CD133+ cells from multiple regions of adult mouse brain, including the cerebellum but were unsuccessful (data not shown). While technical considerations may explain this result, an alternate possibility is that cultures in prior studies included a mixture of Prom1 positive stem/progenitor cells from the SVZ/VZ rather than being exclusively derived from pure white matter *Prom1^hi^* cells, an idea supported by recent studies [29]. To this point, our *in vivo* BrdU labeling shows that *Prom1^hi^* cells in non-germinative regions are slow cycling progenitors but mechanical brain lesioning and irradiation did not appear to stimulate or ablate *Prom1^hi^* cells as has been described for NSCs in the SVZ (Fig. S2) [44]. These observations are interesting given that recent studies in different organ systems (e.g. retina, prostate and hematopoiesis) have also highlighted the non-stem cell expression and function of Prominin-1 [19]–[21]. Our results do not rule out the possibility that *Prom1^h^* ^i^ cells are quiescent non-cycling stem cells that retain the capability to re-enter the cell cycle and self renew and in fact such cells have been recently described with alternative methods and represent an interesting cell type for future study [29], [30].

Our studies and those of others clearly demonstrate that Prom1 is present in multiple glial cell types (ependymal cells, oligodendrocytes, and astrocytes) and suggest that significant differences may exist between mice and humans [45]. Generally CD133 antigen has been less effective as a cancer stem cell marker in mice than humans, possibly related to the differences we have found in the cells expressing Prom1. Within species we also noted differences in the Prom1 cells detected by RNA and protein tools, most likely due to differential recognition of the many Prom1 splice forms and modifications. While some aspects of the differences may be due to technical limitations of RNA in situ hybridization and delayed expression of protein detected by IHC analysis, the diversity of isoforms and modifications to Prom1 imply that such differences more likely do reflect fundamentally diverse functions of Prom1. Corbeil et al. describe splice variant 1 (s1) as dominating in the immature brain and variant 3 (s3) as restricted to postnatal myelinating oligodendrocytes [32]. Although *Prom1^hi^* cells in mouse may be related to the oligodendroglial lineage, the retention of such cells in Olig1/2 knock-out mice, suggests they are not typical oligodendroglial or myelinating cells. Cycling NG2+Olig2+ adult oligodendrocyte progenitors are abundant in the adult brain but the lower BrdU labeling in *Prom1^hi^* cells and lack of co-staining for NG2 distinguishes them from this population. Further studies and new antibody tools capable of recognizing diverse forms of Prom1 will be needed to determine the exact nature of this intriguing Prom1+ cell population and its relation to other known cell types.

As in the normal brain, our results suggest a reassessment of the role for PROM1 in CNS cancers beyond that of a highly specific stem cell marker often assumed in this setting. While PROM1 is ubiquitously referred to as a marker expressed in only a minor subpopulation of stem cells in GBM [14], our studies find this is not consistently the case across different patients. Using TCGA primary GBM data and PDCL data we did not detect a strong association of PROM1 with other markers of stem cells when examined across multiple patients. This included use of methods for RNA expression profiling, RISH, and IHC. However, independent of its stem cell associations, PROM1 seems to be associated with clinically relevant biology in GBM. Multiple studies have shown neurosphere formation or CD133 antigen expression to be associated with shorter survival in patients and mice transplanted with such cells [16], [46], [47]. Our work with *PROM1* RNA and protein as biomarkers agrees with these findings. Despite PROM1 having a poor prognostic value as a cancer stem cell marker, our finding that *PROM1* expression is anti-correlated with IDH1 mutations supports a different biology for IDH mutant tumors and indicates that PROM1 expression may be most relevant in the non-IDH mutant setting. Tools for reliable IHC evaluation of PROM1 in clinical GBM samples are still lacking, but may be useful for further study of these clinical associations.

## Supporting Information

Figure S1 **Prom1+ cells are located in the white matter and ependymal layer of the adult mouse brain.** A. Immunohistochemical analysis for Prom1 shows weak staining restricted to ciliated cells in the ependymal layer. B. The different regions used in the western blot are illustrated in the micrograph. C. Western blot analysis confirms that Prom1 levels are highest in white matter and ependymal layer. D. Prom1+ cells isolated from the mouse cortex analyzed by FACS after MACS sorting show a moderate level of Prom1 protein. E. FACS sorting for CD133 after cleanup by Precoll gradient show a high level of Prom1 in the white matter, which confirmed the RISH staining. cc: corpus callosum; lv: lateral ventricle; svz: subventricular zone; ep: ependymal layer; wm: white matter; gm: grey matter; m: meninges.
(TIF)

Figure S2 ***Prom1^hi^*** **cells in the adult mouse brain do not proliferate in response to stab wound lesion.** A. 7 and 14 days after stab lesion through the cortex and corpus callosum, no *Prom1^hi^*/BrdU cells are detected around the lesion. B. Quantification of the *Prom1^hi^* and Olig2 positive cells around the lesion show no difference compared to contra lateral side control.
(TIF)

Figure S3 ***Prom1^hi^*** **cells in the corpus callosum exhibit relative radioresistance.** *Prom1^hi^* cells 1 and 5 days after 3 Gy of irradiation shows no decrease in the *Prom1^hi^* cell population, while Ki67 proliferative cells are clearly reduced in the subventricular zone (SVZ).
(TIF)

Figure S4 **GBM PDCLs (** ***in vitro*** **) and PDXs (** ***in vivo*** **) harbor heterogeneous PROM1 patterns.** PROM1 immunohistochemistry (A) and FACS (B) on GBM PDCLs and PDXs identify three distinct groups of PROM1 expression, high, medium and low/none. In PDCLs with the highest PROM1 levels all cells express PROM1 homogenously (99% by FACS). PROM1 is however more heterogeneously expressed in PDCLs with a medium (80% by FACS) and low (3% by FACS) PROM1 protein level.
(TIF)

Figure S5 **Immunofluorescence analysis from matching GBM PDX (A) and PDCL (B) (from** **Figure 5B** **) shows that PROM1 cells also stain for stem/glial markers SOX2 and GFAP, few of them being in a proliferative stage, Ki67 positive.**
(TIF)

Figure S6 ***PROM1*** **expression is associated with poor survival and is anti-correlated with IDH1 mutations.** A. *PROM1* expression values in TCGA and GBM PDCLs. The expression value cut offs were arbitrarily designed as followed, <300 =  low, >300 and <1000 =  medium, >1000 =  high. B. PDXs with high expression of Prom1 have a poor overall survival. C. Low expression of *PROM1* correlates with IDH1 mutation in the proneural subclass. D. Proneural TCGA cases with high PROM1 expression do not correlate with age at first diagnosis (r = 0.19).
(TIF)

Table S1 **Probe values of** ***Prom1*** **expression in the GBM PDCLs.**
(XLSX)
