# Supplementary figures and images for: Prominin-1 (CD133) Defines Both Stem and Non-Stem Cell Populations in CNS Development and Gliomas
Source: PLoS One. 2014 Sep 3;9(9):e106694. doi: 10.1371/journal.pone.0106694 (PMC4153667; doi:10.1371/journal.pone.0106694)

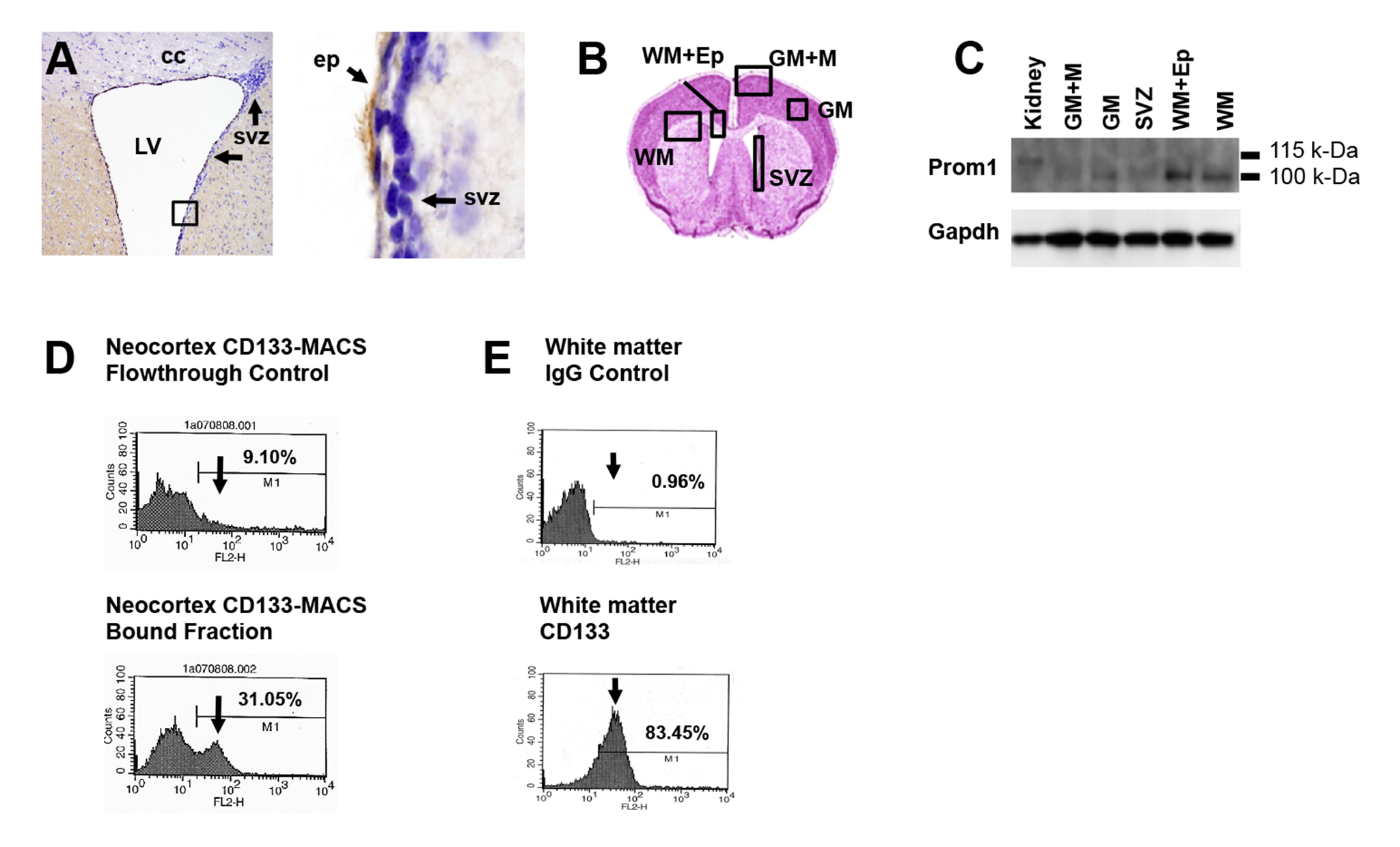

Supplement: Figure S1 — Prom1+ cells are located in the white matter and ependymal layer of the adult mouse brain. A. Immunohistochemical analysis for Prom1 shows weak staining restricted to ciliated cells in the ependymal layer. B. The different regions used in the western blot are illustrated in the micrograph. C. Western blot analysis confirms that Prom1 levels are highest in white matter and ependymal layer. D. Prom1+ cells isolated from the mouse cortex analyzed by FACS after MACS sorting show a moderate level of Prom1 protein. E. FACS sorting for CD133 after cleanup by Precoll gradient show a high level of Prom1 in the white matter, which confirmed the RISH staining. cc: corpus callosum; lv: lateral ventricle; svz: subventricular zone; ep: ependymal layer; wm: white matter; gm: grey matter; m: meninges. (TIF) [file pone.0106694.s001.tif]

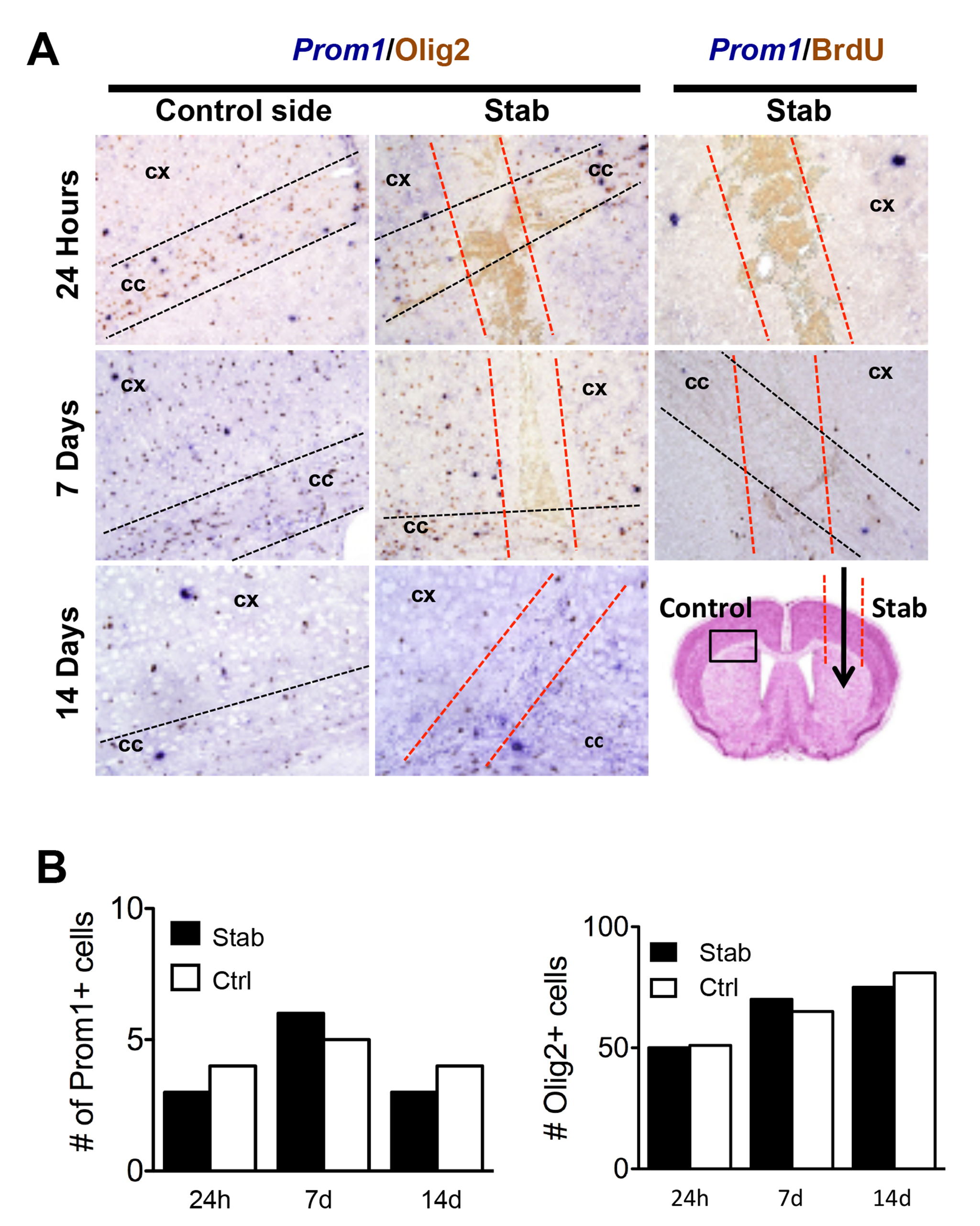

Supplement: Figure S2 — Prom1hi cells in the adult mouse brain do not proliferate in response to stab wound lesion. A. 7 and 14 days after stab lesion through the cortex and corpus callosum, no Prom1hi/BrdU cells are detected around the lesion. B. Quantification of the Prom1hi and Olig2 positive cells around the lesion show no difference compared to contra lateral side control. (TIF) [file pone.0106694.s002.tif]

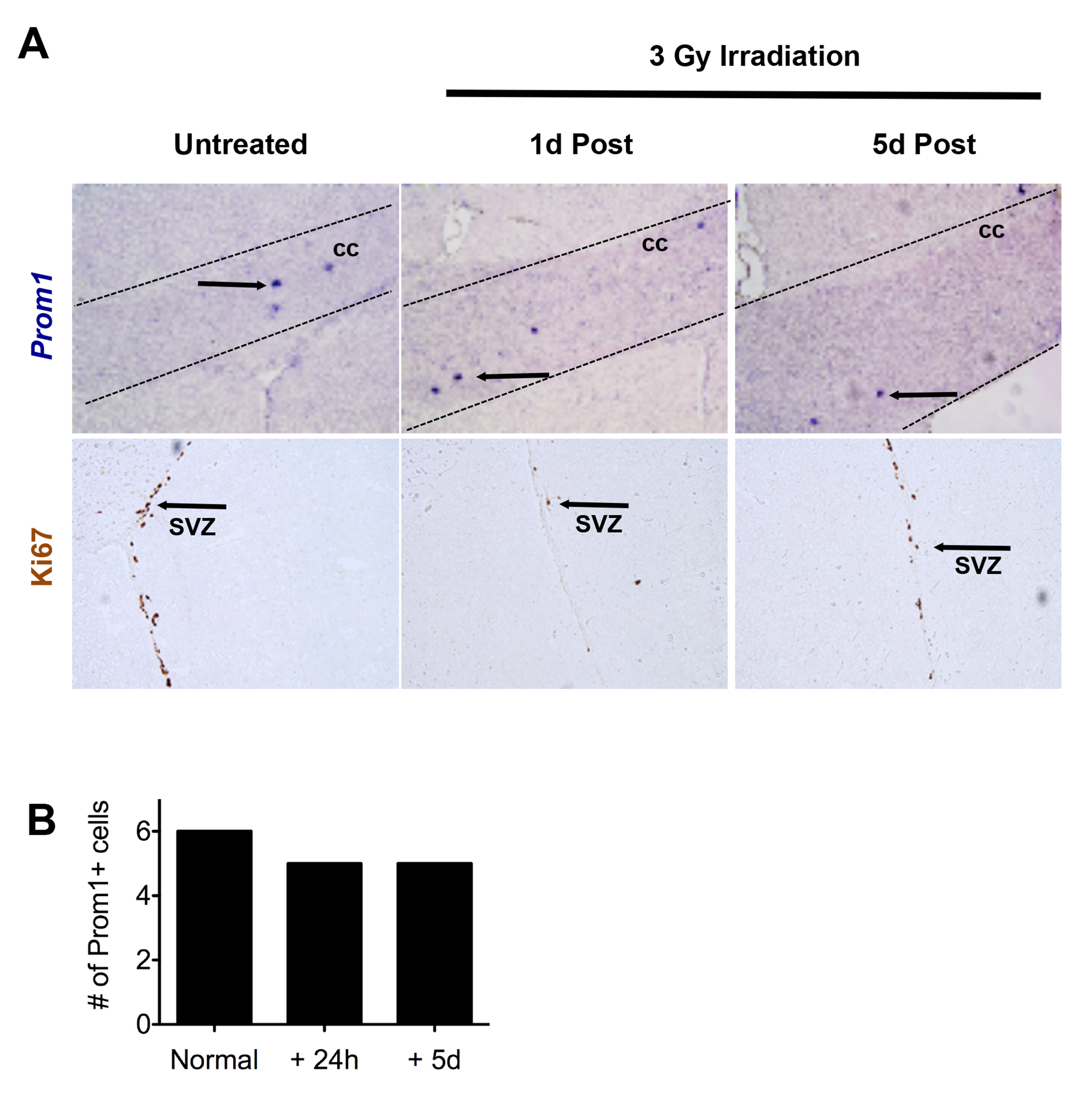

Supplement: Figure S3 — Prom1hi cells in the corpus callosum exhibit relative radioresistance. Prom1hi cells 1 and 5 days after 3 Gy of irradiation shows no decrease in the Prom1hi cell population, while Ki67 proliferative cells are clearly reduced in the subventricular zone (SVZ). (TIF) [file pone.0106694.s003.tif]

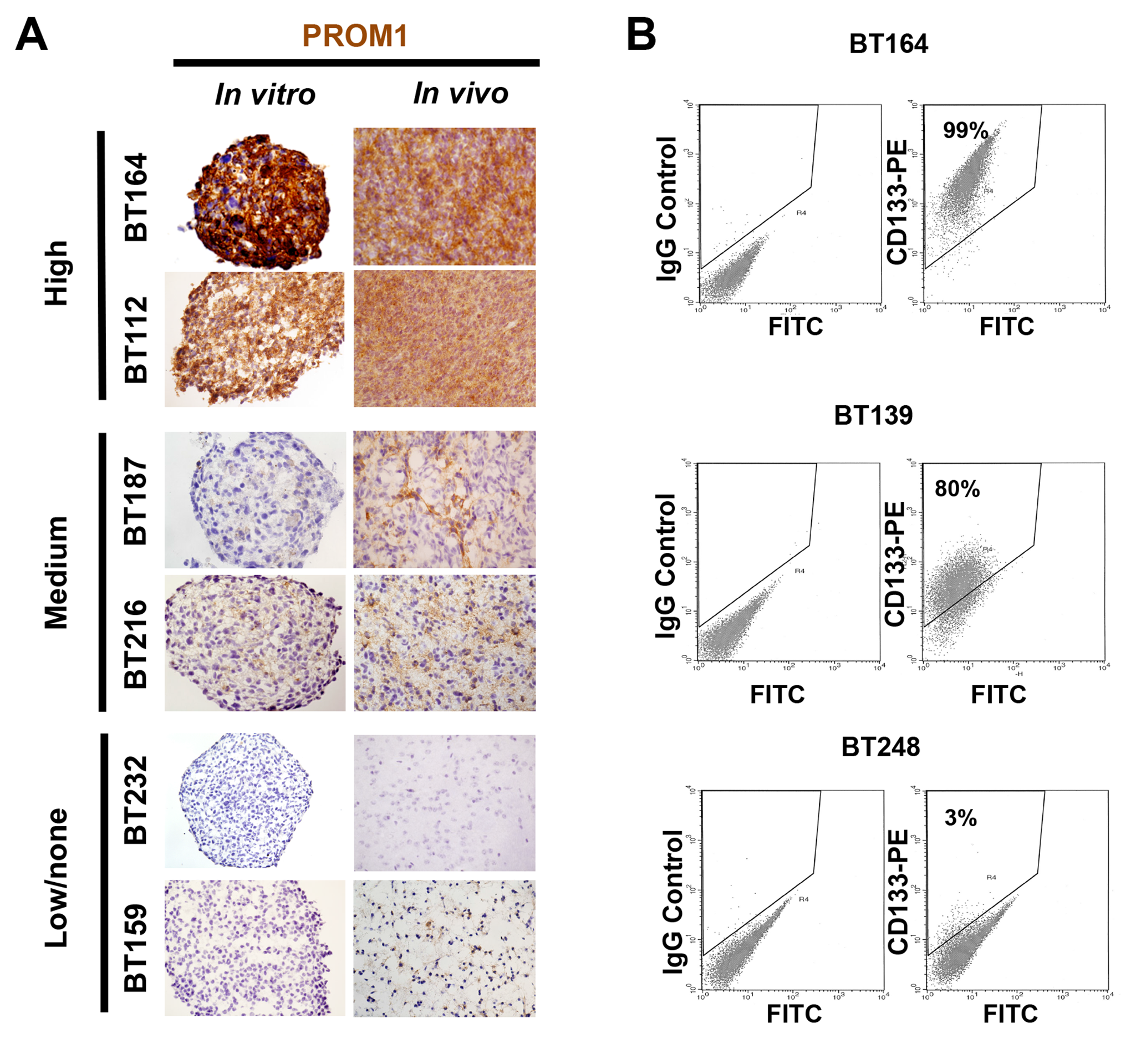

Supplement: Figure S4 — GBM PDCLs ( in vitro ) and PDXs ( in vivo ) harbor heterogeneous PROM1 patterns. PROM1 immunohistochemistry (A) and FACS (B) on GBM PDCLs and PDXs identify three distinct groups of PROM1 expression, high, medium and low/none. In PDCLs with the highest PROM1 levels all cells express PROM1 homogenously (99% by FACS). PROM1 is however more heterogeneously expressed in PDCLs with a medium (80% by FACS) and low (3% by FACS) PROM1 protein level. (TIF) [file pone.0106694.s004.tif]

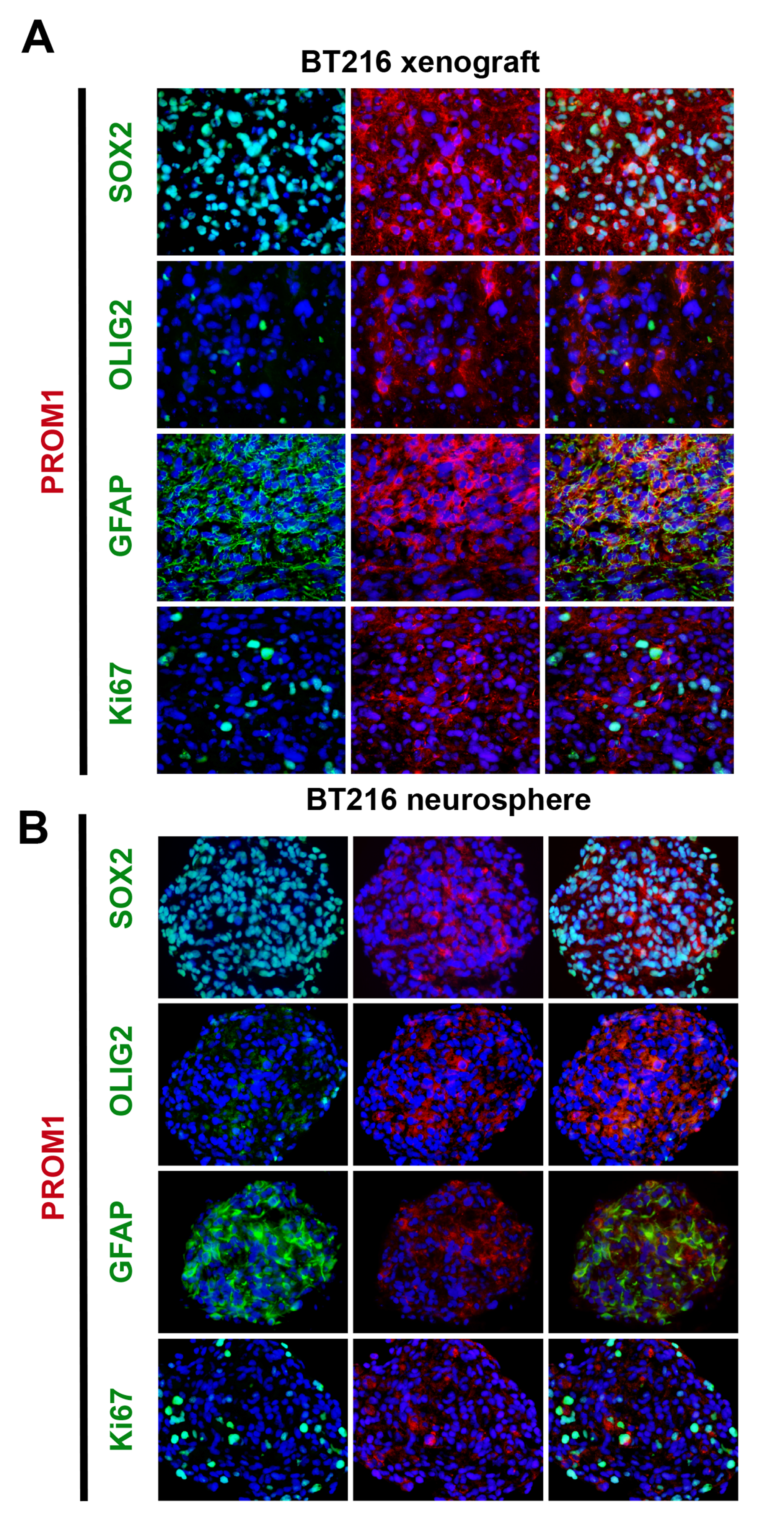

Supplement: Figure S5 — Immunofluorescence analysis from matching GBM PDX (A) and PDCL (B) (from Figure 5B ) shows that PROM1 cells also stain for stem/glial markers SOX2 and GFAP, few of them being in a proliferative stage, Ki67 positive. (TIF) [file pone.0106694.s005.tif]

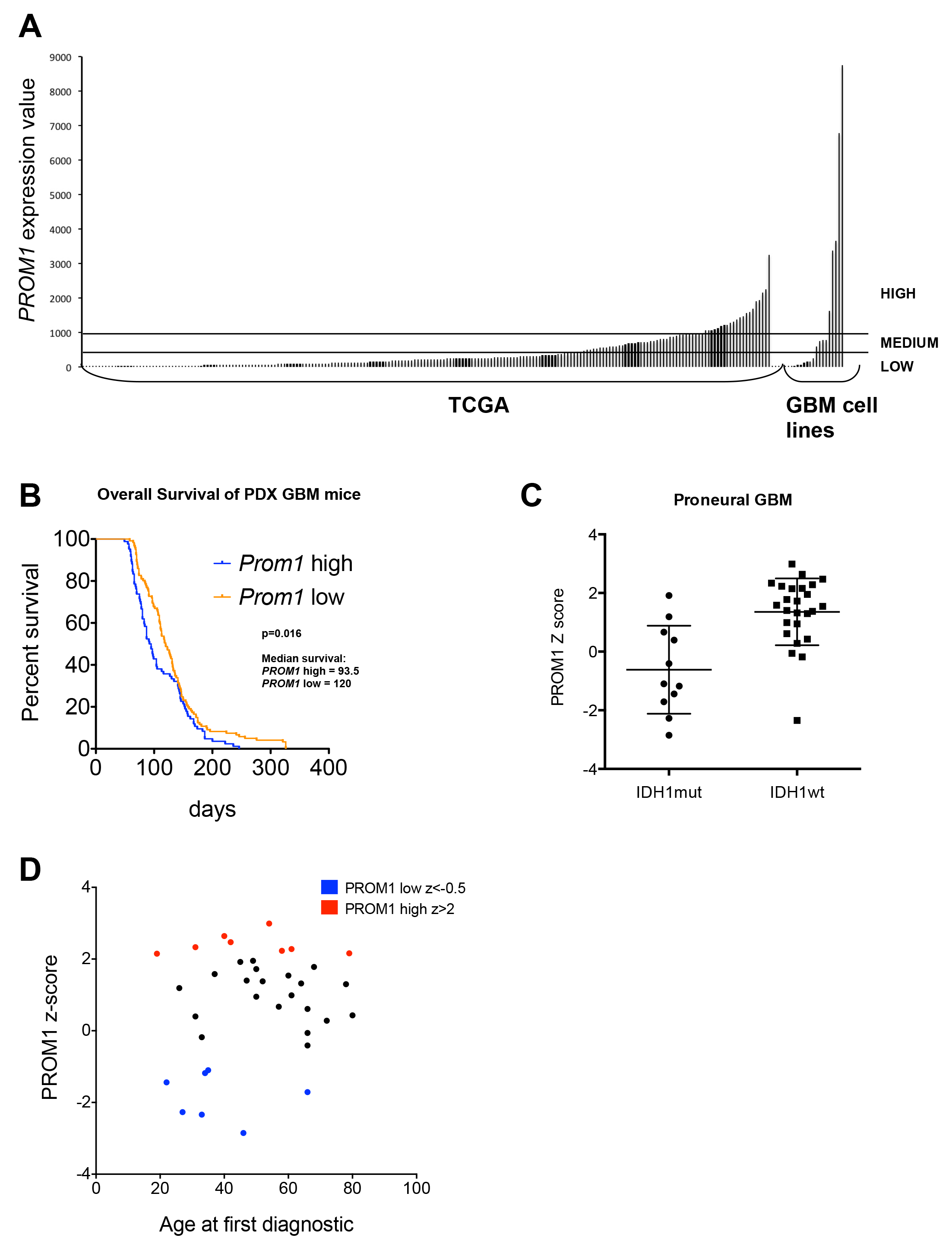

Supplement: Figure S6 — PROM1 expression is associated with poor survival and is anti-correlated with IDH1 mutations. A. PROM1 expression values in TCGA and GBM PDCLs. The expression value cut offs were arbitrarily designed as followed, <300 = low, >300 and <1000 = medium, >1000 = high. B. PDXs with high expression of Prom1 have a poor overall survival. C. Low expression of PROM1 correlates with IDH1 mutation in the proneural subclass. D. Proneural TCGA cases with high PROM1 expression do not correlate with age at first diagnosis (r = 0.19). (TIF) [file pone.0106694.s006.tif]
